# Whole Body Muscle Activity during the FIFA 11+ Program Evaluated by Positron Emission Tomography

**DOI:** 10.1371/journal.pone.0073898

**Published:** 2013-09-16

**Authors:** Junsuke Nakase, Anri Inaki, Takafumi Mochizuki, Tatsuhiro Toratani, Masahiro Kosaka, Yoshinori Ohashi, Junichi Taki, Tetsutaro Yahata, Seigo Kinuya, Hiroyuki Tsuchiya

**Affiliations:** 1 Department of Orthopaedic Surgery, Graduate School of Medical Science Kanazawa University, Kanazawa, Japan; 2 Department of Nuclear Medicine/Biotracer Medicine, Graduate School of Medical Science Kanazawa University, Kanazawa, Japan; 3 Kanazawa Advanced Medical Center, Kanazawa, Japan; The University of Chicago, United States of America

## Abstract

**Purpose:**

This study investigated the effect of the FIFA 11+ warm-up program on whole body muscle activity using positron emission tomography.

**Methods:**

Ten healthy male volunteers were divided into a control group and a group that performed injury prevention exercises (The 11+). The subjects of the control group were placed in a sitting position for 20 min and 37 MBq of ^18^F-fluorodeoxyglucose (FDG) was injected intravenously. The subjects then remained seated for 45 min. The subjects of the exercise group performed part 2 of the 11+for 20 min, after which FDG was injected. They then performed part 2 of the 11+for 20 min, and rested for 25 min in a sitting position. Positron emission tomography-computed tomography images were obtained 50 min after FDG injection in each group. Regions of interest were defined within 30 muscles. The standardized uptake value was calculated to examine the FDG uptake of muscle tissue per unit volume.

**Results:**

FDG accumulation within the abdominal rectus, gluteus medius and minimus were significantly higher in the exercise group than in the control group (P<0.05).

**Conclusion:**

The hip abductor muscles and abdominal rectus were active during part 2 of the FIFA 11+ program.

## Introduction

Prevention of sports injuries has become a key issue in sports medicine in recent years. Most sports injury prevention training programs are composed of plyometric training, balance training, and agility training. Studies have been conducted on the effects of such training programs on various athletes. Although the subjects and details of the training programs differed, the results showed a decreased incidence of sports injuries regardless of sport activity level, sex, and age [1]–[4].

The “11” is an injury prevention program that was developed with the support of the Fēdēration Internationale de Football Association (FIFA) and aims to reduce the effect of intrinsic injury risk factors in soccer. This program has been validated in that sport [5], [6]. A successive modified version of the “11” (the “11+”) has also proven effective in preventing injuries in young female soccer players [7] and elite male basketball players [8]. The FIFA 11+provided a >40% reduction in the risk of injury. Furthermore, research using motion analytic techniques has been conducted on the effect of the sports injury prevention training [9], [10]. The training program aimed to improve core stability and neuromuscular control [11], [12]. However, the muscle activation patterns have not yet been well elucidated for the “11+.”

Muscle activity levels of various sport types have been investigated using electromyographic (EMG) examinations [13], [14]. Since equipment must be attached to the body for EMG measurements, sports activity level is disturbed, which limits the types of sports investigated. In addition, only a limited number of muscles and superficial muscles can be investigated by EMG examinations.

Muscle activity during exercise has been examined by positron emission tomography (PET) with ^18^F-fluorodeoxyglucose (FDG) [15]–[17]. ^18^F-FDG taken up by muscle cells is not metabolized and remains in the cells as FDG-6-phosphate after phosphorylation. Thus, ^18^F-FDG accumulation in the muscle can be used as a parameter of glucose intake by the muscle as well as the muscle activity level. PET provides a promising alternative or supplement to existing methods to assess muscle activation in complex human movements.

The purpose of the present study was to examine muscle activity during the 11+using PET.

## Methods

Ten healthy men volunteered for this study. Five of them were asked to perform the 11+. The 11+consisted of 3 parts: a running exercise (part 1); 6 exercises with 3 levels each of increasing difficulty that developed strength, balance, muscle control, and core stability (part 2); and advanced running exercises (part 3). We intended for the part 2 exercise to consist of level 1 activities except the running exercises (Table 1). Subject characteristics are presented in Table 2. None of the subjects was taking any medications and all were healthy as judged by their medical history and physical examination. The purpose and potential risks of this study were explained to the subjects and written informed consent to participate was obtained from them. The study design was approved by the ethics committee of Kanazawa University Hospital.

**Table 1:** The FIFA 11+ Part 2, Level 1.

Exercise: Strength, plyometric and balance
The bench: Static (30s, 3 sets )
Sideways bench: Static (30s, 3 sets on each side )
Hamstrings: Beginner (3 repetition )
Single-leg stance: Hold the ball (30s, 2 sets )
Squats: With toe raise (30s, 2 sets )
Jumping: Vertical jump (30s, 2 sets )

Exercises of the structured warm-up program.

**Table 2:** Physical characteristics of the subjects in the control and exercise groups (values are mean ± SD).

	control group	exercise group	P value
No. of subjects	5	5	
Age, yr	29±4	31±4	0.30
Height, cm	170.4±4.6	169.4±5.1	0.75
Weight, kg	69.6±9.9	66.4±2.7	0.50
Body mass index,kg/m^2^	23.9±3.0	23.2±1.1	0.64

All subjects refrained from eating and drinking for at least 6 h before the investigation as well as strenuous physical activity for at least 1 day before the experiment.

The subjects in the control group were placed in a sitting position for 20 min and 37 MBq of FDG was then injected intravenously. The subjects then remained seated for 45 min. The subjects in the exercise group performed part 2 of the 11+for 20 min, followed by injection with FDG. Immediately after the injection, each subject performed 20 min of part 2 of the 11+. After resting and exercising, each subject was placed in a supine anatomical position on a scanner bed that facilitated longitudinal displacement into the gantry of a PET-computed tomography (PET-CT) system (Discovery PET/CT 690; GE Healthcare, Milwaukee, WI, USA). The plasma glucose level of each subject was confirmed to be normal before the FDG injection.

Scanning was performed with a 60-cm axial field of view and a transaxial resolution of 6.4 mm (full-width half-maximum in the center field of view without scattering medium). Before emission scanning, an unenhanced CT scan was performed for attenuation correction and muscle orientation. Emission scanning was performed in 3-demensional mode 50 min after ^18^F-FDG administration at 3 min/bed station. The total emission time was 39–42 min. Images were reconstructed with 3-dimensional ordered subset expectation maximization with 2 iterations and 16 subsets. After reconstruction, a 6.4-mm FWMH Gaussian post-filter was applied.

### PET Analysis

Regions of interest (ROI) were drawn manually in 5 areas of the body and 30 skeletal muscles as follows: 1) Trunk: at the inferior border of the fourth lumbar vertebrae for the abdominal rectus as well as for the abdominal external oblique, abdominal internal oblique, transverse abdominal, greater psoas, lumbar quadrate, and erector spinae muscles; 2) Pelvis: at the superior border level of the acetabular roof for the gluteus maximus as well as at the gluteus medius, gluteus minimus, and piriformis muscles; 3) Thigh: at the center of the inferior border of the femoral lesser trochanter, the femoral condyle for the quadriceps femoris muscle, the sartorius, gracilis, semimembranosus, semitendinosus, and biceps femoris muscles, and the adductor muscle complex; 4) Lower leg: at the center of the tibia for the anterior tibial muscle as well as the long flexor muscles of the toes and the great toe and the posterior tibial, triceps surae, and peroneus muscles; 5) Foot: at the center of the navicular for the abductor hallucis muscle, the center of the metatarsal bone for the interosseous muscles, and the plantar quadrate, flexor digitorum brevis, abductor digiti minimi, and flexor hallucis brevis muscles.

One experienced nuclear medicine specialist (A.I.) defined all of the ROI using plain CT images. The standardized uptake value (SUV) was calculated by overlapping of the defined ROI and fusion images. Large vessels were avoided when the muscle areas were outlined. The SUV was calculated to quantitatively examine the FDG uptake of the muscle tissue per unit volume according to the equation: SUV = mean ROI count (cps/pixel)/body weight (g)/injected dose (mCi) × calibration factor (cps/mCi). ROI were defined for the right and left sides of the aforementioned skeletal muscles. The mean SUV was calculated using the following equation: mean SUV = (left mean SUV × left muscle area+right mean SUV × right muscle area)/(left muscle area+right muscle area).

### Statistical Analysis

All data are presented as means and standard deviations. The Mann-Whitney’s U test was used to evaluate differences in muscle volumes and SUV for all ROI between groups. SPSS for Windows ver. 19.0 (SPSS Inc, Chicago, IL, USA) was used for the analysis. The minimum significance level was set at P<0.05.

## Results

**Figure 1:**
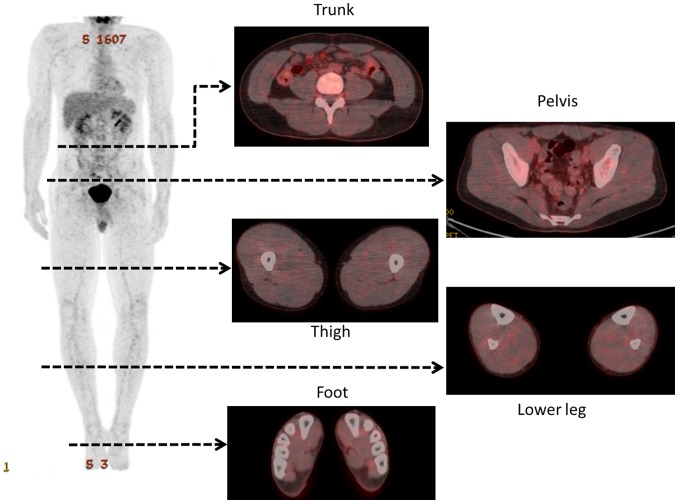
Representative whole-body positron emission tomography images of patients in the control group.

**Figure 2:**
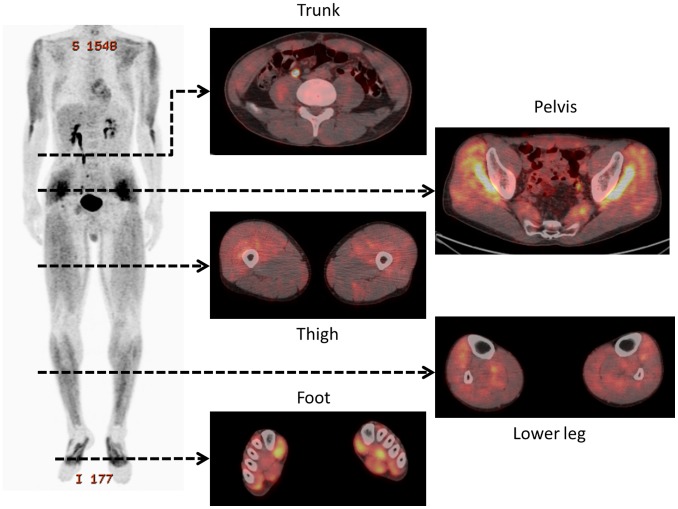
Representative whole-body positron emission tomography images after performance of the 11+ by patients in the exercise group.

No significant differences in individual physical characteristics were observed between groups (Table 2). Figures 1 and 2 illustrate typical whole-body PET images from the control and exercise groups, respectively. Tables 3 and 4 show the ROI volumes and SUVs of the muscles of patients in the control and exercise groups, respectively. No significant differences in ROI volumes were observed between groups for any of the muscles except the flexor hallucis brevis. FDG accumulation within the abdominal rectus, gluteus medius and minimus muscles in the exercise group was significantly higher than that in the control group (P<0.05).

**Table 3:** ROI volumes and mean SUVs in the control and exercise groups, trunk-thigh (values are means ± SD).

		ROI volumes			Mean SUVs		
Body areas	Muscle	control group	exercise group	P value	control group	exercise group	P value
Trunk	Abdominal rectus	11.17±1.32	12.33±3.29	0.50	0.45±0.10	0.82±0.16	***<0.05***
	Abdominal external oblique	17.15±2.75	18.45±4.23	0.58	0.48±0.06	0.62±0.14	0.08
	Abdominal internal oblique	16.43±5.38	19.03±4.89	0.45	0.61±0.06	0.66±0.17	0.54
	Transverse abdominal	7.34±1.65	7.99±1.52	0.54	0.60±0.10	0.59±0.14	0.88
	Greater psoas	24.77±4.70	26.71±5.34	0.56	0.81±0.09	0.80±0.12	0.88
	Lumbar quadrate	14.67±2.96	15.44±3.04	0.70	0.62±0.10	0.55±0.18	0.21
	Erector spinae	41.13±7.40	39.45±7.36	0.73	0.77±0.08	0.66±0.03	0.03
Pelvis	Gluteus maximus	66.14±8.54	67.41±6.37	0.80	0.62±0.05	0.81±0.27	0.21
	Gluteus medius	51.97±9.41	53.74±9.27	0.77	0.76±0.03	1.88±0.27	***<0.05***
	Gluteus minimus	28.69±4.69	22.82±4.80	0.09	0.93±0.14	3.47±0.68	***<0.05***
	Piriformis	16.55±2.13	13.72±4.92	0.29	1.07±0.12	1.44±0.50	0.18
Thigh	Quadriceps femoris	137.74±12.54	129.97±18.66	0.47	0.62±0.09	0.97±0.30	0.06
	Sartorius	5.63±1.95	6.82±2.63	0.44	0.53±0.03	0.51±0.10	0.7
	Gracilis	6.64±1.62	7.37±2.89	0.64	0.47±0.06	0.70±0.26	0.11
	Semimembranosus	16.51±2.23	19.48±7.38	0.43	0.54±0.04	0.57±0.02	0.16
	Semitendinosus	15.29±3.58	16.43±4.85	0.68	0.46±0.07	0.68±0.26	0.13
	Biceps femoris	24.58±4.32	26.96±5.95	0.49	0.51±0.04	0.53±0.05	0.63
	Adductor complex	47.32±7.10	45.84±11.03	0.81	0.63±0.06	0.67±0.07	0.34

ROI, region of interest; SUVs, standardized uptake values.

**Table 4:** ROI volumes and mean SUVs in the control and exercise groups, lower leg-foot (values are mean ± SD).

		ROI volumes			Mean SUVs		
Body areas	Muscle	control group	exercise group	P value	control group	exercise group	P value
Lower leg	Anterior tibial	10.18±0.72	11.75±2.19	0.19	0.78±0.06	0.79±0.21	0.92
	Long flexor muscle of toes	4.24±1.12	4.23±1.48	0.99	0.85±0.41	0.88±0.35	0.91
	Posterior tibial	12.28±1.64	11.41±1.56	0.42	0.92±0.14	1.19±0.52	0.31
	Long flexor muscle of great toe	5.46±1.18	7.13±1.29	0.07	1.06±0.29	1.49±0.49	0.14
	Peroneus	5.49±1.85	4.69±1.11	0.43	0.68±0.09	1.14±0.40	0.06
	Triceps surae	42.51±7.21	39.78±10.05	0.64	0.99±0.40	1.09±0.47	0.71
Foot	Abductor hallucis	7.79±2.86	6.18±1.20	0.29	0.86±0.25	1.55±0.64	0.08
	Plantar quadrate	4.79±0.85	4.83±0.95	0.94	0.92±0.10	1.07±0.30	0.34
	Flexor digitorum brevis	5.55±098	6.36±1.03	0.24	0.84±0.05	1.19±0.49	0.18
	Abductor digiti minimi	5.82±0.86	7.02±1.15	0.10	0.78±0.09	1.22±0.76	0.26
	Flexor hallucis brevis	6.43±0.92	4.40±0.78	*<0.01*	0.84±0.09	1.77±1.02	0.11
	Interosseous	10.88±1.41	9.08±1.06	0.06	0.88±0.12	1.60±0.95	0.17

ROI, region of interest; SUVs, standardized uptake values.

## Discussion

The most important findings of the present study were that the hip abductor and abdominal rectus were active while the patients performed the FIFA 11+injury prevention exercises. To our knowledge, this is the first study to apply PET to the 11+program. These data will be useful in future sports injury prevention programs.

Studies have reported that sports injury prevention programs including the 11+, help reduce the incidence of sports injuries [7], [8]. However, it remains unknown how skeletal muscles throughout the body become active during such programs, since studies to date have primarily used surface EMG to evaluate skeletal muscle activities. Thus, skeletal muscles throughout the body could not be evaluated simultaneously. In our study, PET was used to simultaneously evaluate a variety of skeletal muscles. Our study was the first to demonstrate the activation of the hip abductor, abdominal rectus muscles after the FIFA 11+program.

Fujimoto [15] and Tashiro [18] used PET to evaluate muscle activity during running. These were the first reports of the study of muscle activity during exercise using PET. Other studies have investigated tissue glucose uptake with PET in simple tasks such as isometric muscle contractions [19] and dynamic strength exercises [20] as well as in more complex endurance work tasks such as walking [21], running [22], and double poling [17]. Bojsen et al. reported that PET imaging might be a promising supplement or an alternative to more traditional methods for investigating muscle use during complex human movements [17]. These studies reported that glucose uptake of skeletal muscle up to 55% VO2max intensity closely reflected muscle activity assessed by PET. The present study confirmed that the main effector muscles in the 11+included the hip abductor, abdominal rectus muscles.

Myer et al. [11] reported that hip abduction strength and control might be the critical modulator between altered trunk control and the ultimate lower limb knee loads responsible for sports injuries, especially anterior cruciate ligament (ACL) tears. Russell et al. suggested that the higher valgus angles might predispose women to a higher incidence of ACL injuries [23]. Recent investigations indicate an association of hip abduction strength with dynamic valgus alignments that increase ACL injury risks. Schmitz et al. reported increased gluteus medius activation in response to trunk displacement [24]. Hip abduction strength and recruitment may improve the ability of female athletes to increase lower limb alignment control and decrease motion and loads that result from increased trunk displacement during sports activities [25], [26]. The findings in these reports support the importance of hip abductor and trunk muscle control in preventing sports injuries.

One limitation of the present study is that the PET with FDG method accounts only for muscle glucose uptake and other substrates such as free fatty acids, muscle glycogen, and lactate are metabolized in the active muscle cells. Nonetheless, studies have confirmed that glucose oxidation increases with exercise intensity, and glucose uptake increases, to some extent, in proportion to glycogen utilization when exercise intensity rises [27].

Another limitation of this study was the method used to define ROI. Since FDG uptake was measured at an arbitrary site on the target muscle, it did not reflect that of the entire muscle. In addition, there could have been differences in the glucose uptake ability among skeletal muscle types. In studies to date, FDG uptake has been shown to be higher in the soleus and vastus medialis muscles, which are composed mostly of type 1 fibers, compared to muscles that are composed of type II fibers [28]. It will be necessary to further investigate this issue in future studies. In our study, the daily activities were not restricted in patients of the exercise or control groups on the day of the PET examination. Thus, walking during daily activity could have resulted in a lack of differences in FDG accumulation in the skeletal muscles of the lower legs and feet. Although there are the aforementioned limitations, this study was the first to reveal the effects of the FIFA 11+on muscles throughout the body. The results of this study could provide valuable information to further advance sports injury prevention programs.

## Conclusion

The present study confirmed that the hip abductor and abdominal rectus were active during part 2 of the FIFA 11+. The results shown here could provide valuable information to further advance sports injury prevention programs.
